# Synthetic Protein-Assisted Co-Assembly of Zeolitic Imidazolate Framework-8 and *Novosphingobium capsulatum* for Enhanced Saline–Alkali Resistance of Wheat

**DOI:** 10.3390/molecules30183669

**Published:** 2025-09-09

**Authors:** Zirun Zhao, Rou Liu, Jiawen Yu, Yunlong Liu, Mingchun Li, Qilin Yu

**Affiliations:** National Key Laboratory of Intelligent Tracking and Forecasting for Infectious Diseases, College of Life Sciences, Nankai University, Tianjin 300071, China; 1120240822@mail.nankai.edu.cn (Z.Z.);

**Keywords:** ZIF-8, betaine, *Novosphingobium*, rhizosphere targeting, saline-alkali soil

## Abstract

Soil saline–alkali stress is a major problem faced by global agriculture, and there is an urgent need to develop efficient amelioration strategies. While both probiotics and plant stress-resistant molecules play critical roles in the alleviation of crop stress, their efficient retention in crop rhizosphere regions remains a great challenge. In this study, the nanocarrier ZIF-8@SPBP@betaine (ZSBet) was constructed by introduction of the synthesized polysaccharide-binding protein (SPBP) and the stress-resistant molecule betaine to the metal–organic framework ZIF-8. During co-incubation, the probiotic *Novosphingobium capsulatum* and ZSBet efficiently bound together to form ZSBet + Novo co-assemblies, i.e., the integrated protein-ZIF-8-probiotic complexes mediated by polysaccharide-receptor recognition, which exhibited strong root-binding abilities. Microbiome analysis revealed that ZSBet + Novo reduced the α-diversity of rhizosphere bacteria and increased the absolute abundance of biofilm formation-related bacteria, e.g., *Novosphingobium*, *Sphingobium*, and *Lactococcus*. During wheat cultivation in saline–alkali soil, ZSBet + Novo reduced soil pH by 0.63 units, decreased soil salt content by 0.11 g/kg, and increased soil nutrient levels. Furthermore, the co-assembly enhanced the wheat grain number by 145.05% and reduced root malondialdehyde and proline contents by 42.00% and 39.13%, respectively. This study provides a new strategy for improving crop resistance under saline–alkali stress in combination with nanotechnology and synthetic biology.

## 1. Introduction

Soil salinization and alkalization are two of the main forms of global soil degradation at present, which seriously reduces the area of arable land and threatens the sustainable development of global agriculture [1,2]. Saline–alkali stress interferes with the root functions of plants through various mechanisms such as osmotic stress, oxidative damage, ionic toxicity, and alkaline-induced metabolism dysfunction [3,4,5]. Moreover, the stress inhibits photosynthesis, limits the absorption of water and nutrients, and thereby hinders the normal growth of plants [6,7,8]. Although traditional methods such as physical drainage-based salt washing, chemical neutralization, and salt-tolerant crop breeding have alleviated saline–alkali stress to a certain extent [9,10,11,12], there are generally problems such as poor stability, high input costs, and a delayed onset of effectiveness [13,14]. Therefore, it is becoming the key direction to develop sustainable and eco-friendly amelioration strategies for improving crop stress resistance and production during utilization of the saline–alkali soils.

Among the emerging amelioration strategies, rhizosphere probiotics have received extensive attention due to their important role in regulating the saline–alkali resistance of plants [15,16]. In particular, extracellular polysaccharide (EPS)-producing microbes can form protective biofilms, isolate harmful ions, and modulate rhizosphere pH and redox homeostasis, thereby contributing to a more stable microecological environment [17,18]. Meanwhile, small molecule osmotic protectants, such as betaine, proline, salicylic acid, and flavonoids [19,20,21], are also widely used in the regulation of plant stress resistance due to their functions in regulating cell osmotic pressure, protecting enzyme activity, activating the Na^+^/H^+^ transport system, and scavenging reactive oxygen species [22,23,24]. However, in practical applications, these two types of strategies still have challenges: rhizosphere probiotics are often difficult to be stably implanted due to the lack of effective anchoring sites [25,26,27], and exogenous protective agents are also prone to leaching or rapid degradation [28]. Therefore, it is urgently necessary to construct an integrated platform that can not only achieve the effective targeted delivery of protective molecules but also promote the stable colonization and synergistic effect of rhizosphere probiotics.

A metal–organic framework (MOF) is a kind of porous nanomaterial and has become an important carrier for the transportation of agricultural active substances. Their high specific surface area, adjustable pore structure, surface functionalized plasticity, and excellent sustained-release performance make them particularly suitable for penetrating protective agents and plant hormones and other stress-relieving molecules in challenging soil environments [29,30,31]. In particular, zinc-based MOF ZIF-8 constructed from Zn^2+^ and 2-methylimidazole has other advantages in agricultural applications, including biocompatibility, hydrolytic stability, and mild synthesis conditions [32,33,34]. Recent studies have shown that ZIF-8 can support pH-responsive or enzyme-triggered release of active molecules, improving the stability and utilization efficiency of compounds in the soil environment [35]. Despite these advantages, most MOFs lack biological targeting [36]. Therefore, precise targeting strategies have been extensively explored in the biomedical field, where surface functionalization of MOFs with ligands such as folic acid, peptides, or antibodies enables efficient and selective recognition of specific tumor cells or biomolecules [37,38,39]. In contrast, comparable functionalization approaches aimed at plant roots and their associated beneficial rhizosphere microbes have rarely been reported. When applied to the soil, they cannot selectively anchor to the roots of plants or beneficial bacteria, which limits their functioning in the soil microenvironment under abiotic stress. To overcome this challenge, functional biological components need to be integrated into the MOF system so that the system can target to the rhizosphere.

To address the challenges of complex soil stress, this study aims to construct an assembly that integrates the sustained release of substances, the synergy of rhizosphere probiotics, and their joint targeting of the rhizosphere. Betaine is a mature osmotic protectant [40,41]. Due to its role in maintaining cellular osmotic balance and stabilizing enzyme activity [42,43,44], betaine was selected as the stress-resistant molecule in this experiment. Meanwhile, the polysaccharide-rich surface of plant roots and the extracellular polymers of probiotics have become the keys to designing targeted proteins. To address this challenge, we functionalized ZIF-8, which remains stable in the saline–alkali condition, with the synthetic polysaccharide-binding protein (SPBP, molecular weight = 38.3 kD, Figure S1) containing the carbohydrate-binding domain of *Trichoderma reesei* Cel6A (CBD_Cel6A_), and further loaded the plant stress-resistant molecule betaine, obtaining the co-assembled nanocarrier ZIF-8@SPBP@betaine (ZSBet). We hypothesize that SPBP can promote dual anchoring (root and microbes), and together with sustained betaine release, increase salt tolerance. Our findings reveal that ZSBet efficiently colonizes the rhizosphere, mitigates saline–alkali stress, reshapes the rhizosphere microbiome, and significantly improves wheat stress resilience. This work lays a technological foundation for the development of next-generation strategies for soil remediation.

## 2. Results

### 2.1. Characterization of the Synthesized ZIF-8

**Figure 1:**
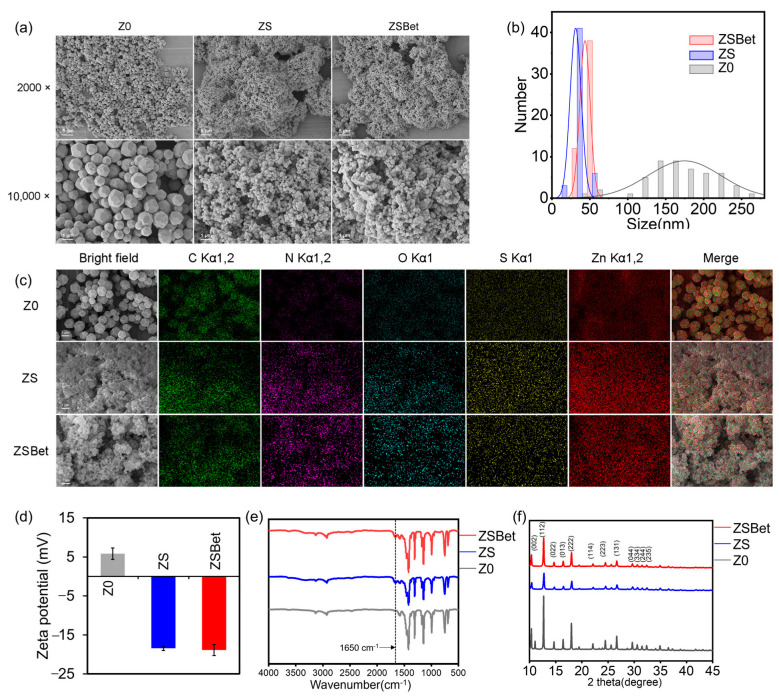
Characterization of the synthesized Z0 (pristine ZIF-8), ZS (SPBP-functionalized ZIF-8), and ZSBet (SPBP-functionalized ZIF-8 loaded with betaine). (**a**) Scanning electron microscopy (SEM) observation of the synthesized ZIF-8. (**b**) Size distribution of the synthesized ZIF-8 revealed by dynamic light scattering (DLS) analysis. (**c**) Energy-dispersive spectroscopy (EDS) elemental mapping images of the synthesized ZIF-8. Green, purple, blue, yellow, and red represent carbon, nitrogen, oxygen, sulfur, and zinc, respectively. (**d**) Zeta potentials of the synthesized ZIF-8. (**e**) Fourier-transform infrared (FTIR) spectra of the synthesized ZIF-8. The gray curve represents Z0, the blue curve represents ZS, and the red curve represents ZSBet. (**f**) X-ray diffraction (XRD) of the synthesized ZIF-8. The gray curve represents Z0, the blue curve represents ZS, and the red curve represents ZSBet.

In recent years, nanomaterials have demonstrated great potential in the agricultural field [45,46,47]. In particular, ZIF-8 has shown promise in enhancing crop nutrition and improving stress resistance [48]. Z0 represents the conventional ZIF-8 nanoparticles synthesized from zinc nitrate and 2-methylimidazole [49]. However, such conventional ZIF-8 exhibits a critical limitation. It has difficulty achieving efficient rhizosphere colonization, thus preventing its full potential from being realized. To address this issue, the study introduced a synthetic polysaccharide-binding protein (SPBP), previously developed by our team. This protein simultaneously recognizes and recruits both natural extracellular polysaccharide-producing microbes and plant root tissues. A modified material, designated ZS, was obtained by covalently incorporating the synthetic polysaccharide-binding protein (SPBP) during the synthesis of Z0. Building upon this, ZSBet was prepared by further loading the plant-derived stress-resistance molecule betaine onto ZS, with the betaine-encapsulating efficiency at 82 mg/g. Scanning electron microscopy (SEM) revealed that Z0 formed well-defined dodecahedral crystals with sharp edges and uniform particle size distribution (Figure 1a). In contrast, incorporation of SPBP (ZS) and subsequent loading of betaine (ZSBet) accelerated nucleation kinetics, resulting in smaller particles with pronounced aggregation (Figure 1a). Dynamic light scattering (DLS) analysis corroborated these findings (Figure 1b, Figure S2), which showed a mean hydrodynamic diameter of 174.67 ± 48.01 nm for Z0, whereas ZS and ZSBet exhibited substantially reduced sizes of 31.33 ± 7.37 nm and 43.62 ± 6.79 nm, respectively. This marked decrease in particle sizes of ZS and ZSBet, which may be attributed to the inhibition of further growth by the added SPBP during ZIF-8 synthesis, are theoretically favorable for increasing the specific surface area [50]. Energy-dispersive X-ray spectroscopy (EDS) mapping indicated comparable carbon (C) distribution and content across all three samples (Figure 1c). Notably, ZS and ZSBet displayed elevated levels of nitrogen (N), oxygen (O), sulfur (S), and zinc (Zn) relative to Z0. The enrichment of N, O, and S elements results from the incorporation of SPBP in ZS and the synergistic presence of SPBP and betaine in ZSBet.

Zeta potential measurements revealed a significant shift in surface charge: Z0 exhibited a slightly positive surface potential (+5.86 mV), whereas ZS and ZSBet showed strongly negative values (−18.40 mV and −18.84 mV, respectively) (Figure 1d). This shift is consistent with the incorporation of acidic functional groups, such as carboxyl (-COOH), introduced by SPBP and betaine. Fourier-transform infrared (FTIR) spectroscopy revealed a distinct absorption peak at 1650 cm^−1^ for ZS and ZSBet, absent in Z0 (Figure 1e). This feature corresponds to the C=O stretching vibration of amide bonds in SPBP, confirming successful protein conjugation. X-ray diffraction (XRD) patterns confirmed high crystallinity in all materials (Figure 1f), with diffraction peaks aligning with standard ZIF-8 references [51]. The retention of these peaks in ZS and ZSBet indicates that the framework structure remained intact after SPBP and betaine incorporation. Moreover, after 110 days of storage in air, Z0 retained the characteristic reflections of the ZIF-8 framework, while the peaks of ZS (Z0 + SPBP) and ZSBet (Z0 + SPBP + Betaine) were significantly weakened (Figure S3), indicating a gradual transformation of the modified frameworks into an amorphous state.

The betaine-releasing kinetics of ZSBet at different pH and NaCl concentrations were further investigated. As shown in Figure S4, while ZSBet released quite low levels of betaine, 6.1% and 5.8% at the pH values of 7 and 9 after 48 h of incubation, the nanocarrier rapidly released betaine, 29% in 8 h, and remained almost stable with longer time (Figure S4a). Moreover, the nanoparticles only released low levels of the cargo (<6%) at different concentrations of NaCl (Figure S4b). These results suggested that the particles may retain betaine with a long time in the saline–alkali condition.

### 2.2. The SPBP-Mediated Binding and Co-Assembly of ZS and ZSBet with Probiotics

During the interaction between rhizosphere probiotics and plant roots, extracellular polymers (EPS) secreted by the probiotics played a key role in alleviating plant salt stress [52]. EPS can promote the formation of biofilms, participate in osmotic regulation (for example, by reducing the sodium ion activity of microbial cells or rhizosphere), and contribute to the reorganization of microbial community composition [53,54,55]. However, some probiotics, e.g., *Novosphingobium capsulatum* (Novo), have the problem of a low rhizosphere colonization ability. In response to this issue, this study explored the interaction between metal–organic framework materials (ZIF-8 variants Z0, ZS, and ZSBet) and the Novo cells, aiming to evaluate whether these materials could enhance the rhizosphere colonization ability of this strain.

**Figure 2:**
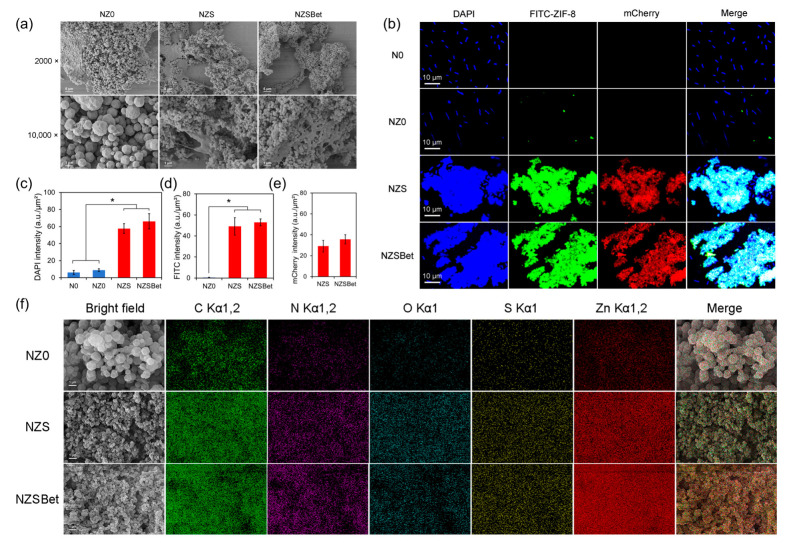
The interaction between synthetic ZIF-8 variants (i.e., Z0, ZS, ZSBet) and the probiotic bacterium *Novosphingobium capsulatum* (Novo). (**a**) SEM observation of the interaction of synthetic ZIF-8 variants with Novo. (**b**) Confocal laser scanning microscopy images of the interaction of synthetic ZIF-8 with Novo. DAPI (blue) stains the nuclei of Novo cells; FITC-ZIF-8 (green) indicates FITC-labeled ZIF-8 nanoparticles; and mCherry (red) corresponds to the synthesized protein SPBP. (**c**) Quantification of the DAPI fluorescence. (**d**) Quantification of the FITC fluorescence. (**e**) Quantification of the mCherry fluorescence. (**f**) Energy-dispersive spectroscopy (EDS) elemental mapping images of the interaction of synthetic ZIF-8 with polysaccharide-producing bacteria. Green, purple, blue, yellow, and red represent carbon, nitrogen, oxygen, sulfur, and zinc, respectively. The asterisks (*) indicate significant differences between the groups (*p* < 0.05).

Scanning electron microscopy (SEM) was first employed to examine the attachment behavior of Novo upon co-incubation with Z0, ZS, and ZSBet (designated as NZ0, NZS, and NZSBet, respectively) (Figure 2a). SEM images show that bacterial adhesion on the surface of Z0 is scarce. In sharp contrast, both ZS and ZSBet showed dense bacterial colonization on their surfaces and formed a unique reticular biological aggregate structure similar to biofilm. To further visualize and quantify these interactions in real time, confocal laser scanning microscopy (CLSM) was employed. Z0, ZS, and ZSBet were pre-labeled with fluorescein isothiocyanate (FITC), and bacterial nucleic acids were stained with DAPI. Notably, the SPBP functionalized on ZS and ZSBet also provided intrinsic mCherry fluorescence. The experimental groups were as follows: N0 (Novo cells only), NZ0 (FITC-Z0 + Novo), NZS (FITC-ZS + Novo), and NZSBet (FITC-ZSBet + Novo). After 10 min of co-incubation (Figure 2b–e), CLSM revealed a low spatial overlap between FITC and DAPI signals in the NZ0 group, suggesting the poor binding affinity of Z0 toward bacterial cells. In contrast, both NZS and NZSBet groups showed strong FITC fluorescence, tightly enclosing bacterial aggregates. More importantly, a strong co-localization phenomenon was observed among FITC (material), DAPI (Novo), and mCherry (SPBP) signals, indicating that ZS and ZSBet could form co-assembly structures with bacteria while effectively recruiting them. Energy-dispersive X-ray spectroscopy (EDS) analysis further confirmed that compared with NZ0, the contents of microbial-related elements (C, O, N and S) in NZS and NZSBet, as well as the zinc content of the materials themselves, were significantly increased (Figure 2f). In summary, it was demonstrated that the SPBP-functionalized metal–organic framework material was able to efficiently recruit Novo cells and promote the formation of stable nanocarrier–probiotic co-assemblies. Meanwhile, ZSBet, even at 200 mg/L, had no obvious impact on the viability of Novo cells (Figure S5), suggesting good biocompatibility of the particles to the probiotics.

### 2.3. The SPBP-Mediated Root Targeting of ZS and ZSBet Co-Assemblies with Probiotic Bacteria

To evaluate the binding ability of the probiotic Novo cells to the root system of wheat (*Triticum aestivum* L.) in the presence of the MOF-based materials Z0, ZS, and ZSBet, we conducted co-culturing experiments of the root system, materials, and rhizosphere probiotic, and characterized them by SEM and EDS. Four experimental groups were established: RCK (root + Novo only), RZ0 (root + Z0 + Novo), RZS (root + ZS + Novo), and RZSBet (root + ZSBet + Novo).

**Figure 3:**
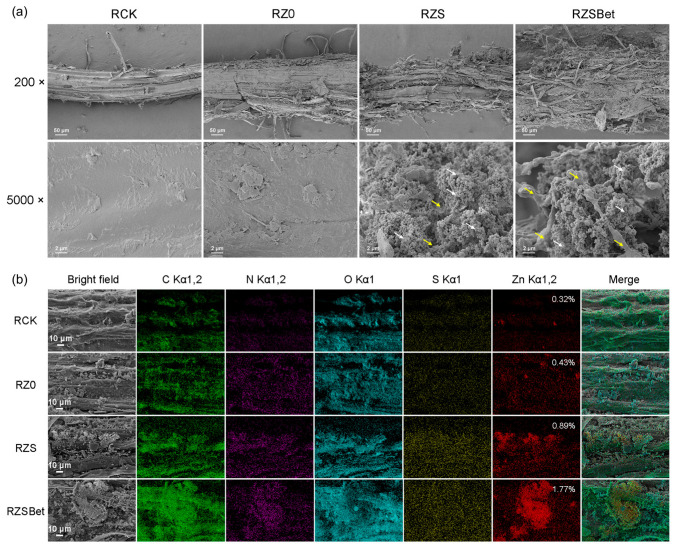
Colonization patterns of ZIF-8 variants and the probiotic *Novosphingobium capsulatum* (Novo) on wheat roots under saline–alkali stress. The treatments included Z0 (pristine ZIF-8), ZS (SPBP-functionalized ZIF-8), ZSBet (SPBP-functionalized ZIF-8 loaded with betaine), and Novo inoculation. (**a**) Scanning electron microscopy (SEM) images of the treated wheat roots. The yellow arrows indicate representative probiotic cells, while the white arrows indicate the ZIF-8 materials. (**b**) Energy-dispersive spectroscopy (EDS) elemental mapping images show the distribution of C, N, O, S, and Zn on the root surfaces. Green, purple, blue, yellow, and red correspond to C, N, O, S, and Zn, respectively.

SEM imaging at both low (200×) and high (5000×) magnifications revealed distinct differences in material colonization across groups (Figure 3a). Among them, the root surface structures of the RCK and RZ0 groups were relatively smooth, and almost no obvious bacterial aggregation or material deposition was observed, indicating that the bare MOF material Z0 itself had difficulty achieving effective colonization. In contrast, the root surfaces of the RZS and RZSBet groups showed extensive material adsorption and bacterial adhesion phenomena, forming a dense network structure-like biofilm, demonstrating typical microbial–material co-assembly characteristics. Consistently, colony-forming unit (CFU) assays revealed that the roots treated by RZS and RZSBet captured much more bacteria than the roots treated by RCK and RZ0 (1.54 × 10^7^~2.08 × 10^7^ CFU/g roots versus 0.17 × 10^7^~0.30 × 10^7^ CFU/g roots, Figure S6). Elemental mapping via EDS further corroborated these findings (Figure 3b). Compared with RCK and RZ0, the signal intensities of microbial-related elements (such as C, N, O, and S) in RZS and RZSBet were significantly enhanced. Meanwhile, RZS and RZSBet exhibited distinct Zn signals derived from the MOF materials. Intensity quantification further showed that higher levels of Zn signals appeared on the roots of the RZS and RZSBet groups than that on the roots of the RCK and RZ0 groups (26,695~36,694 versus 2554~5002, Figure S7). These results indicated that the ZIF-8 materials and probiotics achieved more efficient co-enrichment and colonization under the effect of SPBP. In conclusion, the introduction of SPBP significantly enhanced the attachment and enrichment ability of MOF materials on the root surface, promoted the co-colonization and co-assembly of probiotics and materials, and thus realized the potential for targeted rhizosphere localization.

### 2.4. The Co-Assembly of ZSBet and Novo Modifies the Rhizosphere Microbiome

**Figure 4:**
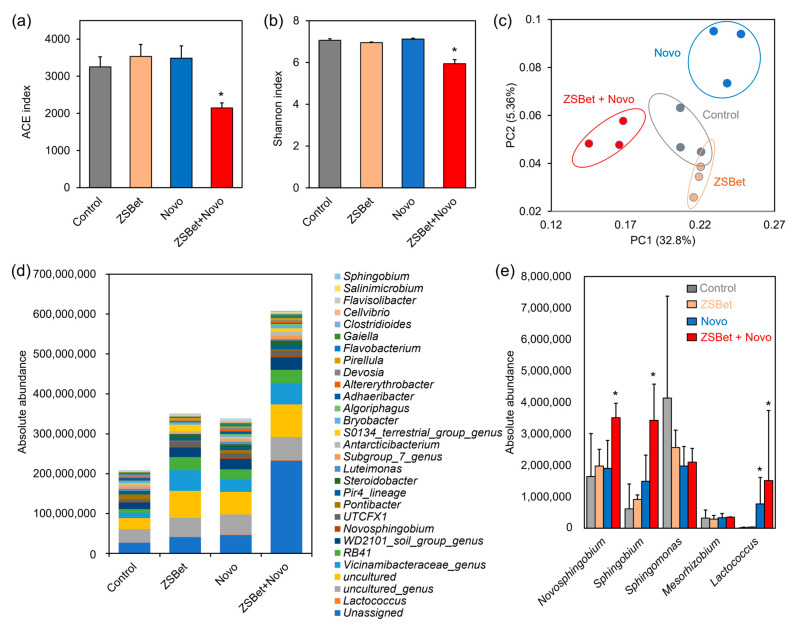
Microbiome analysis of the wheat rhizosphere soils by 16S rDNA sequencing in the four experimental treatments, i.e., the control (untreated), the ZSBet (ZIF-8@SPBP@betaine nanocarrier), the Novo (*N. capsulatum* inoculation), and the ZSBet + Novo (combined treatment). (**a**) Abundance-based coverage estimator (ACE) index of the four groups. (**b**) Shannon index of the four groups. (**c**) Principal coordinate analysis (PCA) of the microbiome composition in the four groups with the weighted-unifrac method. (**d**) Absolute abundance of the bacterial groups between the treatments at the genus level. Spike-in read counts were used to generate a standard curve for the abundance analysis. Total reads = 8,666,393. FDR < 0.05. (**e**) Statistical analysis of polysaccharide-producing bacterial genera under different treatments. The asterisks (*) indicate significant differences between the groups (*p* < 0.05).

To evaluate the potential of ZSBet and Novo in ameliorating saline–alkali soils, we established four treatments: Control, ZSBet, Novo, and the combined ZSBet + Novo treatment during wheat cultivation. After 110 days, rhizosphere soil samples were collected from the wheat rhizosphere regions and subjected to total DNA extraction for high throughput 16S rDNA sequencing. The results revealed that the application of the ZSBet + Novo co-assembly significantly influenced the structure and diversity of rhizosphere microbial communities, while ZSBet or Novo alone had no significant impact on the diversity compared with the control (Figure 4a,b). Specifically, the α-diversity indices, including ACE and Shannon, were significantly reduced under the ZSBet + Novo treatment, indicating a marked decline in both species’ richness and community evenness (Figure 4a,b). This reduction in diversity is likely attributable to the enhanced colonization of functional bacteria (e.g., Novo). Such colonization may further promote competitive exclusion and microenvironmental restructuring, thereby suppressing hazardous microbial populations.

Principal component analysis (PCA) further confirmed these trends. The samples of each treatment group showed obvious clustering, with small differences within the groups and significant differences between the groups (Figure 4c). In terms of bacterial species composition, both ZSBet and Novo treatments significantly increased the overall absolute abundance of rhizosphere bacteria, while the combined treatment further induced a synergistic expansion effect of bacterial populations (Figure 4d). Speciation analysis at the genus level revealed that the combined treatment significantly upregulated the abundance of three typical polysaccharide-producing genera, including *Novosphingobium*, *Lactococcus*, and *Sphingobium* (Figure 4e). These bacteria have a strong ability to secrete extracellular polysaccharides (EPSs), which can not only promote the formation of rhizosphere biofilms [56,57,58], but also help binding to the SPBP in ZSBet materials, thereby achieving effective anchoring and stable colonization of the co-assembly on the root surface. The enrichment of such polysaccharide-producing bacteria helps to construct a protective rhizosphere microenvironment and enhances the adaptability of plants to saline–alkali stress.

### 2.5. The Co-Assembly of ZSBet and Novo Improves the Quality of Saline–Alkali Soil

To investigate whether shifts in the rhizosphere microbiome could influence the physicochemical properties of saline–alkali soils, we conducted comprehensive soil analyses following 110 days of treatment. Rhizosphere samples were collected from wheat plants and assessed for key soil parameters. The application of the ZSBet + Novo co-assembly resulted in a marked reduction in soil pH and salinity, with pH decreasing from 8.5 to 7.9 and salt contents from 4.3 g/kg to 3.1 g/kg (Table 1). These changes indicate a substantial alleviation of alkaline stress and ionic toxicity by the co-assembly, thereby creating a more favorable microenvironment for root development and nutrient uptake. In parallel, the ZSBet + Novo treatment significantly increased the soil organic matter, total nitrogen, available nitrogen, and available phosphorus to higher levels than ZSBet or Novo alone (Table 1). These improvements reflect enhanced carbon turnover and increased nutrient bioavailability, indicative of improved soil fertility. Collectively, these findings demonstrate that the integrated application of ZSBet and Novo not only improves the physicochemical conditions of saline–alkali soils but also enhances nutrient accessibility.

### 2.6. The Co-Assembly of ZSBet and Novo Boosts Crop Resistance in Saline–Alkali Soil

**Figure 5:**
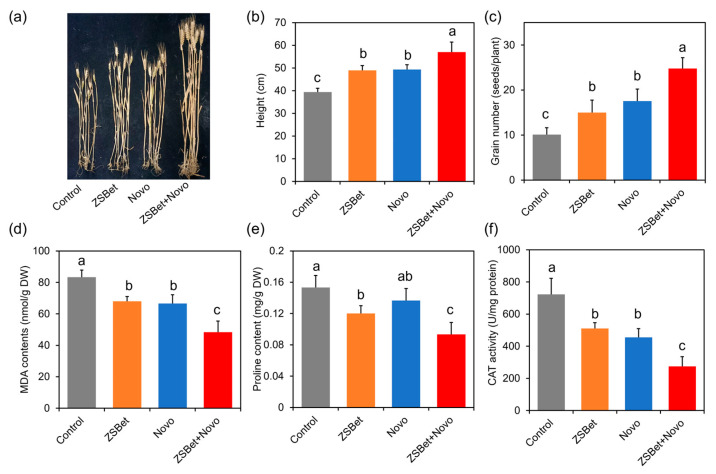
Effects of different treatments (Control, ZS, ZSBet, and ZSBet + Novo) on wheat growth and root physiological characteristics under saline–alkali stress. (**a**) Morphological comparison of wheat plants after 110 days of cultivation. (**b**) Plant height. (**c**) Grain number per spike. (**d**) Root malondialdehyde (MDA) content. (**e**) Root proline (Pro) content. (**f**) Root catalase (CAT) activity. Data are expressed as mean ± standard deviation (SD, *n* = 5). Different lowercase letters indicate statistically significant differences among treatments at *p* < 0.05 (one-way ANOVA followed by Tukey’s test).

Following 110 days of treatment, morphological and physiological assessments of wheat plants were conducted to evaluate the effects of the ZSBet + Novo co-assembly on plant performance under saline–alkali conditions. Both treatments individually led to significant improvements in plant growth, as evidenced by increased plant height and spike number per plant. Moreover, the co-assembly treatment led to the most pronounced effect on wheat growth (Figure 5a–c). These results point to a synergistic interaction between ZSBet and Novo in promoting vegetative development.

To assess plant stress responses, the levels of root malondialdehyde (MDA), a biomarker for lipid peroxidation and oxidative damage, were measured in root tissues. Both ZSBet and Novo reduced MDA accumulation from 83 nmol/g dry weight (DW) to 66–68 nmol/g DW when applied alone. More strikingly, the co-assembly further reduced MDA levels to 48 nmol/g DW (Figure 5d). This indicates a more effective mitigation of oxidative stress in the root zone. Proline, a well-characterized osmoprotectant, functions to maintain cellular osmotic balance, stabilize enzymes, and scavenge free radicals under salt stress [59]. Catalase, a key antioxidant enzyme, detoxifies hydrogen peroxide and prevents excessive reactive oxygen species (ROS) accumulation [60]. Both of them were induced by saline–alkali stress. As shown in Figure 5e,f, the co-assembly markedly reduced concentrations of proline and catalase (CAT) in the roots. The observed reduction in these two markers indicates that the combined treatment alleviated osmotic and oxidative stress in the roots and helped maintain cellular homeostasis. Therefore, the co-assembly can not only promote the morphological growth of plants but also alleviate the impact of saline–alkali stress on plants. This dual function highlights the potential of this comprehensive strategy in improving the performance and stress resistance of degraded agricultural soil crops.

## 3. Discussion

Soil salinity and alkalinity continue to threaten global agriculture, urgently requiring efficient and environmentally sustainable remediation strategies [61,62]. In this study, to address the rapid degradation of stress-responsive small molecules and the limited colonization ability of rhizosphere probiotics, we developed an intelligent nanocarrier named ZSBet. This sustained-release platform was co-assembled from a synthetic polysaccharide-binding protein (SPBP), betaine, and metal–organic framework (ZIF-8), enabling the targeted delivery of the protective osmolyte betaine. ZSBet can be assembled in conjunction with rhizosphere probiotics and precisely targeted at plant roots. This co-assembled system further enhances the adaptability of plants to saline–alkali stress by strengthening rhizosphere colonization, sustainably releasing stress-resistant molecules, and regulating microbial communities, thereby providing an efficient and sustainable solution for saline–alkali soil remediation.

### 3.1. Construction of the Nanocarrier ZIF-8@SPBP@betaine

In recent years, materials such as biochar and synthetic polymers have also been widely studied for soil improvement and nutrient slow release [63,64]. Biochar, due to its high porosity, can improve soil structure, but its nutrient release is usually slow and difficult to regulate, and its performance is greatly affected by raw materials and pyrolysis conditions [65]. Synthetic polymers (such as poly-γ-glutamic acid) have also been explored as carriers, but their biodegradability and stability in soil are limited, which can easily lead to a rapid loss of active components and increase application costs [66].

Compared with these materials, metal–organic frameworks (MOFs), as an efficient nano-sustained-release carrier, offer new solutions to the above-mentioned problems. MOF has been widely applied in the agricultural field for loading fertilizers, pesticides and plant hormones [67]. However, traditional metal-based MOFs (such as those containing iron, zirconium, or chromium) have problems like metal ion toxicity, harsh high-temperature synthesis conditions and high costs, which limit their large-scale promotion and application in agricultural ecosystems [68,69,70]. In contrast, zinc-based metal–organic frameworks (Zn-MOF) have the advantages of mild synthesis conditions and environmental friendliness and are more suitable for agricultural uses. Aiming at the problems that traditional exogenous protective agents are prone to degradation in soil [71] and the MOF system has limited targeting ability with rhizosphere probiotics [72,73], we designed and synthesized a multifunctional nanocarrier ZSBet, which integrates artificial polysaccharide-binding protein (SPBP), ZIF-8, and the anti-osmotic protective agent betaine.

Due to its abundance of polar functional groups (e.g., -COOH, -NH_2_, -SH), SPBP facilitated ZIF-8 nucleation, leading to a significant reduction in particle size (from 174.67 nm to approximately 31–44 nm) (Figure 1b), improved particle uniformity, and increased specific surface area [74]. The resulting ZS and ZSBet materials exhibited negatively charged surfaces, as confirmed by zeta potential measurements, and the presence of SPBP was verified by FTIR (amide I band at 1650 cm^−1^) and EDS mapping (enrichment of N, O, and S elements) (Figure 1c,e). XRD analysis shows that the crystal structure of ZIF-8 remains intact after the introduction of SPBP (Figure 1f). In terms of function, ZSBet is conducive to improving the loading capacity and sustained-release performance of betaine. To evaluate the long-term stability, XRD analyses of Z0, ZS, and ZSBet were performed after 110 days of storage in air (Figure S3). Z0 retained the characteristic reflections of the ZIF-8 framework, while the peaks of ZS (Z0 + SPBP) and ZSBet (Z0 + SPBP + Betaine) were significantly weakened, indicating a gradual transformation of the modified frameworks into an amorphous state. Notably, ZSBet exhibited a more pronounced diffuse peak in the low-angle region, suggesting that the presence of betaine further disrupted the ordered structure, accelerated the amorphization process, and was accompanied by a sustained-release behavior. In addition, we found that Zn^2+^ was partially released from the ZIF-8 materials, with the soil Zn^2+^ contents increasing from 136~138 μg/g soil to 241~256 μg/g soil (i.e., the decomposition rate of ZIF-8 at 10~11%, Figure S8). It is hypothesized that under the saline–alkali conditions, the gradual degradation of ZIF-8 may release Zn^2+^, which may contribute to the enhanced ability of plants to scavenge ROS [75,76,77,78], while its organic ligand component may be degraded by local soil microorganisms [79]. The rational design of SPBP and its successful integration in ZIF-8 nanoparticles provide a solid foundation for constructing an intelligent delivery system with targeting and continuous release functions in plant-related environments.

### 3.2. SPBP Mediates the Co-Assembly of ZIF-8 and Probiotics for Enhancing Their Targeted Localization to Plant Roots

The microbial production of extracellular polymeric substances (EPSs) plays a pivotal role in mitigating plant salt stress by regulating rhizosphere ion balance, promoting biofilm formation, and enhancing osmotic stability [80]. Against this background, our study demonstrated that, compared with the unmodified Z0 material, SPBP-functionalized ZIF-8 materials (ZS and ZSBet) significantly enhanced the recruitment of the EPS-producing bacterium *N. capsulatum* (Figure 2). Furthermore, scanning electron microscopy, energy-dispersive spectroscopy, and confocal microscopy revealed that Zn signals had quite low levels in both the RCK group without MOF and the RZ0 group containing only pristine ZIF-8, whereas Zn enrichment was clearly observed in the RZS group without betaine and the RZSBet group containing betaine and SPBP-modified ZIF-8. Notably, ZSBet formed a dense network structure with the probiotics and further induced the co-assembly of ZSBet and the probiotics without impairing *N. capsulatum* viability (Figure S5). This indicates a synergistic interaction between the particles and the bacterial cells.

In addition to the adhesion of bacteria in vitro, the CBD_Cel6A_ domain in SPBP endows ZSBet with the ability to recognize plant cell wall polysaccharides, thereby achieving effective anchoring of the root surface. The SEM and EDS data further indicated that in the co-culture system of ZS and ZSBet with wheat roots, the adhesion ability of the materials on the root surface was significantly higher than that of Z0. Especially in the RZSBet group, dense co-aggregation of MOF particles, probiotics, and root tissues could be observed. This phenomenon might result from the combined effect of polysaccharide recognition mediated by SPBP and EPS formed by the capsule *N. capsulatum*. Further speculation suggests that the EPS secreted by probiotics can encapsulate the ZIF-8 particles, thereby slowing down their degradation process and enhancing the material adhesion and system stability of the root surface. Meanwhile, the biofilm matrix may form a hypoxic and weakly acidic microenvironment in the rhizosphere, which is conducive to the continuous colonization of salt-tolerant microbial communities [81,82]. In conclusion, the dual functions of SPBP in co-assembly formation and root targeted anchoring are of great significance for maintaining the microbial activity in the rhizosphere under saline–alkali stress conditions.

### 3.3. The Co-Assembly of ZSBet and Novo Reshaped the Rhizosphere Microbial Community in Saline–Alkali Soil

The co-assembly formed by ZSBet and the probiotic *N. capsulatum* (Novo) markedly restructured rhizosphere microbial communities under saline–alkali conditions. The α-diversity indices (ACE and Shannon) decreased slightly, indicating that the species richness and evenness of microorganisms decreased somewhat, but the absolute abundance of bacteria increased significantly. This change may be jointly driven by the SPBP-mediated material–microbial co-assembly and the stable microenvironment constructed by betaine. Community species composition analysis revealed that the relative abundances of genera such as *Novosphingobium*, *Sphingobium*, and *Lactococcus* significantly increased, indicating a gradual transformation of the rhizosphere microecology from random colonization to a deterministic assembly model dominated by polysaccharide-producing bacteria. All these three types of bacteria have the ability to secrete extracellular polymers (EPS), thereby effectively alleviating salt stress. In addition, the genus *Lactococcus* prefers a mild acidic environment (with an optimal pH of 5.5–6.2) [83]. It reduces the local pH of the rhizosphere by secreting organic acids such as lactic acid, which helps alleviate alkaline stress [84]. In conclusion, the co-assembly facilitated the construction of a core microbial community dominated by EPS-producing flora in the rhizosphere, providing stable and favorable microecological support for plants to resist adverse stress.

### 3.4. The Co-Assembly of ZSBet and Novo Promoted the Systematic Improvement in Soil Physicochemical Properties and Crop Stress Resistance

The co-assembly constructed by ZSBet and Novo significantly improved soil properties and crop traits under saline–alkali stress conditions. Analysis of soil physical and chemical indicators shows that the pH value and salinity have decreased significantly, while the contents of organic matter, total nitrogen, and available nutrients have increased significantly. These improvements are attributed to the slow-release effect of betaine in the ZSBet system and the enrichment and activity of the microbial community. The latter can secrete extracellular polysaccharides (EPSs) and organic acids, thereby improving the soil structure and physical and chemical properties. The reconstruction of the rhizosphere microbiota promotes the enrichment of beneficial functional bacterial communities, enhances the biogeochemical cycling function of the soil, and provides a solid ecological foundation for the restoration of saline–alkali land.

At the plant level, the co-assembly significantly promoted the growth of wheat and alleviated salt stress. The decrease in the levels of malondialdehyde (MDA), proline, and catalase (CAT) indicates a reduction in rhizosphere oxidation and osmotic stress, which may be attributed to the osmotic protective effect of betaine [85] and the shielding effect provided by microbial biofilms. The improvement in these physiological indicators, combined with the enhancement of nutrient absorption, helps maintain cellular homeostasis and enhance the overall stress resistance of plants. Together with enhanced nutrient uptake, these physiological changes help maintain cellular homeostasis and improve the overall stress resistance of plants.

**Figure 6:**
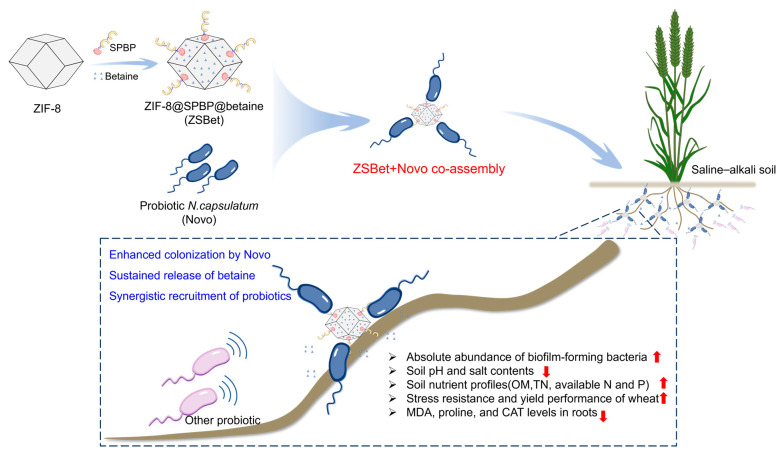
A scheme illustrating the preparation process of ZIF-8@SPBP@betaine, the co-assembly with probiotics, and the regulation of the rhizosphere microbial community through probiotic colonization and betaine sustained release, thereby enhancing the salt tolerance of plants and improving soil quality. SPBP, synthetic polysaccharide-binding protein; OM, organic matter; TN, total nitrogen; MDA, malondialdehyde; CAT, catalase. Red arrows pointing upward indicate horizontal increase, while those pointing downward indicate horizontal decrease.

In summary, the ZSBet + Novo co-assembly not only supports rhizosphere colonization and synergistic microbial interactions but also offers a promising strategy for improving soil quality and crop performance in saline–alkali environments (Figure 6). Nevertheless, several limitations should be noted. The present results are restricted to wheat, and the persistence of the effects across other crops remains to be confirmed. The long-term ecological safety of introducing such a system, particularly its potential influence on native microbial communities, requires further study. Moreover, the economic cost and feasibility of scaling up to large field applications must be considered before broader application. Finally, the relatively small number of replicates in this field study may limit statistical power, and future work should incorporate more replicates and multi-crop trials to strengthen the robustness and applicability of the findings.

### 3.5. Future Perspectives

This study has developed a multifunctional nanotechnology platform for saline–alkali land remediation. The core component, ZSBet, is a targeted controlled-release system based on ZIF-8 with strong structural flexibility and assembly compatibility. It enables tailored delivery strategies for various soil conditions and crop types. Leveraging the porous structure and surface modifiability of ZIF-8, future studies can further incorporate environmental response mechanisms, enabling ZSBet to intelligently sense saline–alkali stress, rhizosphere pH, or root exudates, thereby achieving precise release and temporal control of stress-resistant small molecules and probiotics. Additionally, the co-assembly of ZSBet with the Novo strain can optimize its physical–chemical properties to jointly recruit diverse, functionally complementary microbial communities, thereby enhancing the resilience and stability of the rhizosphere microbiome. In the future, the modular design concept of ZSBet can be extended to more soil degradation types and agricultural application scenarios, providing a replicable materials science solution for sustainable agricultural development. Meanwhile, we also found that Zn^2+^ was partially released from the ZIF-8 materials (Figure S8). Consistently, it was reported that the environmental factors may lead to partial decomposition of ZIF-8, which have diverse impacts, such as the regulation of biological metabolism and cytotoxicity [86,87]. The potential effect of its degradation products on rhizosphere microbiome and plant metabolism will be investigated in future studies.

## 4. Materials and Methods

### 4.1. Materials

Zn(NO_3_)_2_·6H_2_O, 2-methylimidazole, and betaine of an analytical grade were purchased from Aladdin, China. The recombinant SPBP, previously developed in our laboratory, has a molecular weight of 38.3 kDa and consists of CBD_Cel6A_ domains. The sequence of SPBP is shown in Figure S1. It was expressed in the engineered *E. coli* strain *EcSPBP*, purified, and stored in lyophilized form. The amino acid sequence and synthetic pathway are provided in Supplementary Figure S1. All other solvents were of analytical or deionized grade.

### 4.2. Synthesis and Characterization of ZIF-8-Based Materials

To synthesize the Z0 material, 150 mg (i.e., 0.504 mmol) of Zn(NO_3_)_2_·6H_2_O was dissolved in 5 mL of deionized water, and 330 mg (i.e., 4.024 mmol) of 2-methylimidazole was dissolved in 10 mL of methanol. Under magnetic stirring at 200 rpm and room temperature, the zinc nitrate solution was slowly added to the 2-methylimidazole solution and sonicated for 15 min. The resulting precipitate was washed three times with ethanol and deionized water, followed by freeze-drying to obtain the Z0 composite. For the preparation of ZS, the procedure was the same as above, except that 1 mL of recombinant SPBP solution (10 mg/mL) was added after mixing the zinc nitrate and 2-methylimidazole solutions. The mixture was then sonicated for 15 min, washed, and freeze-dried to obtain the ZS composite. For ZSBet synthesis, 5 mL of betaine solution (10 mg/mL, containing 0.426 mmol betaine) and 1 mL of recombinant SPBP solution (10 mg/mL) were sequentially added to the reaction mixture after combining the zinc nitrate and 2-methylimidazole solutions. The mixture was sonicated for 15 min, then washed and freeze-dried following the same procedure to obtain the ZSBet composite.

The physicochemical properties of the nanocomposites were characterized using the following techniques: scanning electron microscopy (SEM, TESCAN MIRA LMS, Brno-Kohoutovice, Czech Republic) for morphological analysis, Fourier-transform infrared spectroscopy (FTIR) for functional group identification, X-ray diffraction (XRD, SmartLab-SE, Rigaku Corporation, Tokyo, Japan) for crystal structure determination, and dynamic light scattering (DLS) along with zeta potential analysis (Zetapals/BI-200SM, Brookhaven, Upton, NY, USA) for particle size distribution and surface charge measurement.

### 4.3. Microbial Recruitment Assay

To evaluate the bacterial adsorption capacity of ZIF-8 materials, *N. capsulatum* was first cultured in liquid Mart medium (composed of 1% glucose, 0.5% peptone, 0.05% KH_2_PO_4_, 0.1% MgSO_4_, 0.05% FeSO_4_, 0.1% vitamin mixture, and 0.1% trace element mixture) at 30 °C with shaking for 12 h. The bacterial cells were then harvested by centrifugation at 12,000 rpm for 2 min and resuspended in phosphate-buffered saline (PBS, pH = 7.2). The cell suspension was adjusted to an optical density of 0.1 at OD_600_. Z0, ZS, and ZSBet were subsequently added to the suspension at a final concentration of 100 mg/L. The mixture was incubated at 25 °C with gentle shaking for 1 h, after which the materials were separated by centrifugation.

A portion of the recovered materials was dehydrated through a graded ethanol series, dropped onto copper grids, freeze-dried, and examined using scanning electron microscopy (SEM) combined with energy-dispersive X-ray spectroscopy (EDS) for morphological and elemental analysis. The remaining portion was used for fluorescence imaging. FITC-labeled Z0, ZS, and ZSBet were co-incubated with *N. capsulatum* pre-stained with DAPI (4,6-diamidino-2-phenylindole) for 30 min. Samples were divided into four groups: N0 (*N. capsulatum* only), NZ0 (FITC-Z0 + *N. capsulatum*), NZS (FITC-ZS + *N. capsulatum*), and NZSBet (FITC-ZSBet + *N. capsulatum*). Notably, ZS and ZSBet exhibited intrinsic mCherry fluorescence due to the presence of SPBP. After two PBS washes, the samples were observed under a confocal laser scanning microscope (Olympus, Tokyo, Japan) for fluorescence imaging analysis.

### 4.4. Root-Binding Assay

*Triticum aestivum* L. were grown in test soil until they reached a height of approximately 10 cm. The soil used was collected from the top layer (0–15 cm) of a wheat field in the Binhai New Area of Tianjin, China. To investigate the colonization ability of the bacterial strain in the rhizosphere under different material treatments, wheat roots were harvested and washed three times with PBS buffer. The cleaned roots were then immersed in a suspension of *N. capsulatum* at an OD_600_ of 0.1, followed by the addition of ZIF-8-based materials (Z0, ZS, or ZSBet) at a final concentration of 100 mg/L. The experiment consisted of four groups: RCK (roots + *N. capsulatum*), RZ0 (roots + *N. capsulatum* + Z0), RZS (roots + *N. capsulatum* + ZS), and RZSBet (roots + *N. capsulatum* + ZSBet). After 24 h of incubation at room temperature, the roots were collected and washed three times with PBS, then fixed in 2% glutaraldehyde solution (*w*/*v*, Sigma, St., Louis, MO, USA) for 24 h. The samples were subsequently dehydrated through a graded ethanol series and dried using a freeze vacuum dryer (10 N, SCIENTZ, Ningbo, China). The dried root samples were observed under a scanning electron microscope (SEM, TESCAN MIRA LMS, Czech Republic), and elemental distribution on the root surface was analyzed using the coupled energy-dispersive X-ray spectroscopy (EDS) system. To quantify the viable bacteria adhered to the roots, the collected roots without fixation were weighed and homolyzed in PBS. The obtained suspensions were diluted by distilled water, followed by addition to the Mart plates. CFUs were counted after 48 h of culturing at 25 °C.

### 4.5. Field Experiment Site and Design

The field experiment was conducted on representative saline–alkali soil in the Binhai New Area of Tianjin, China. The soil conditions of the fields were as follows: pH 8.6, salt content 0.49%, organic matter 25.92 g/kg, TN 1.15 g/kg, available N 75.03 mg/kg, available P 39.51 mg/kg. Four treatment groups were established: Control (no treatment), ZSBet (application of ZSBet only), Novo (inoculation with *N. capsulatum* only), and ZSBet + Novo (combined application of ZSBet and *N. capsulatum*). The fields were equally divided by 20 plots (20 m × 5 m for each plot, length × width), and each treatment was randomly assigned in five replicate plots (*n* = 5). Ridges were established between plots to prevent cross-contamination. Normal irrigation was performed three times. After wheat sowing, the microbial inoculant Novo and ZSBet were applied to the root zone. The Novo suspension was prepared at a concentration of 10^8^ CFU/mL and applied at 10 mL per plant, resulting in an inoculation dose of 10^9^ CFU per plant. Based on the typical field planting density of approximately 120 plants/m^2^, this corresponds to an application density of ~2 × 10^11^ CFU/m^2^. ZSBet material was applied at a dosage of 1 g/m^2^ to achieve rhizosphere anchoring and sustained betaine release. During the entire growth period, all plots were managed uniformly with consistent irrigation and manual weeding to ensure comparable growth conditions and minimize interference from non-treatment factors.

### 4.6. Microbial Diversity Analysis

Rhizosphere soils were sampled after 110 days of cultivation from the control group, ZSBet group, Novo group, and ZSBet + Novo group were analyzed by 16S rDNA sequencing technology, and the composition and abundance of microbial communities were analyzed in combination with absolute quantitative technology. The total DNA was extracted from the soil samples using the FastDNA^®^SPIN kit (MP Biomedicals, Santa Ana, CA, USA). For absolute quantification, synthetic spike-in sequences were designed to retain the conserved regions of the 16S rDNA gene while replacing the variable regions with randomized bases and were added at defined gradient copy numbers prior to PCR. They were co-amplified with the bacterial 16S rDNA gene using primers 515F (5′-GTGCCAGCMGCCGCGG-3′) and 907R (5′-CCGTCAATTCMTTTRAGTTT-3′) and sequenced together with the microbial DNA. Spike-in read counts were used to generate a standard curve for converting OTU/ASV reads into absolute copy numbers, which were normalized to copies per ng of DNA and further adjusted to sample mass (e.g., copies/g soil). Gene copy number correction was applied to estimate microbial cell abundance. Sequencing was performed by Genesky Biotechnologies Inc. (Shanghai, China), and the resulting data were analyzed via the Genesky Biotech Cloud platform (http://cloud.geneskybiotech.com, accessed on 8 June 2025). Sequences were clustered into operational taxonomic units (OTUs) at 97% similarity and denoised using the DADA2 pipeline within the QIIME2 software (version 1.41.1). Taxonomic annotation of bacterial species was conducted using the SILVA database (version 138.1). Alpha diversity was assessed by calculating the ACE and Shannon–Wiener indices. Principal coordinate analysis (PCA) of microbial communities and genus-level abundance analysis were conducted as previously described [27].

### 4.7. Determination of Rhizosphere Soil Physicochemical Properties

After 110 days of cultivation, rhizosphere soil samples were collected from plants under different treatments. The soils were air-dried and passed through a 2 mm sieve. A 10 g subsample was mixed with 50 mL of deionized water, shaken for 30 min, and then filtered. The pH of the filtrate was determined using a pH meter. The electrical conductivity (EC) of the filtrate was measured using a conductivity meter, and the soil salt content (g/kg) was calculated based on a standard curve. Soil organic matter content was determined using the potassium dichromate oxidation method [88]. Total nitrogen content was analyzed using a carbon–nitrogen analyzer (Elemantar Vario EL/macro cube, Elementar Analysensysteme GmbH, Langenselbold, Germany). Soil available nitrogen was measured by the alkaline hydrolysis diffusion method [89]. Available phosphorus content was determined by the molybdenum–antimony anti-colorimetric method [90]. The soil Zn^2+^ contents in the rhizosphere soils of the plants were determined by an inductively coupled plasma optical emission spectrometer (PerkinElmer Optima, PerkinElmer, Rodgau, Germany).

### 4.8. Measurement of Plant Growth and Root Biochemical Indicators

After 110 days of cultivation, the wheat plants reached maturity for measurement of plant growth and biochemical analysis. To measure the plant height and the number of grains per spike, five areas with 1 m × 1 m were randomly chosen for each treatment, and all of plants were sampled for measurement of heights and grain numbers. The average heights and the average numbers of grain per spike were then calculated. For biochemical analysis, six root samples were collected from each plant, with five plants selected per treatment condition (*n* = 5). Proline and malondialdehyde (MDA) contents in root tissues were determined using commercial kits (i.e., the proline content assay kit and the MDA content assay kit, Solarbio Life Sciences, Beijing, China). MDA content was measured following the thiobarbituric acid (TBA) method, where MDA reacts with TBA under acidic and high-temperature conditions to form a red product with an absorption peak at 532 nm. Results were expressed as nmol/g DW. Proline content was determined after extraction with sulfosalicylic acid (SA) and reaction with acid ninhydrin to produce a red compound with an absorption peak at 520 nm. Results were expressed as mg/g DW. Soluble protein in root tissues was determined using the Bradford method with bovine serum albumin (BSA) as the standard. Catalase (CAT) activity was measured with a commercial kit (Solarbio Life Sciences, Beijing, China) following the manufacturer’s instructions. One unit (U) of CAT activity was defined as the amount of enzyme decomposing 1 μmol H_2_O_2_ per minute at 25 °C. The specific activity of CAT was calculated and expressed as U/mg protein.

### 4.9. Statistical Analysis

Each experiment was performed in five replicates (*n* = 5), and data are presented as mean ± standard deviation (SD). All statistical analyses were conducted using SPSS software (version 29, IBM, Armonk, NY, USA). One-way analysis of variance (ANOVA) was used to evaluate differences among treatments, followed by Tukey’s post hoc test for multiple comparisons. Differences were considered statistically significant at *p* < 0.05. For α-diversity indices, multiple comparisons were corrected using the Bonferroni method to control the false discovery rate (FDR), with adjusted *p* < 0.05 regarded as statistically significant.

## 5. Conclusions

In conclusion, we developed the abiotic/biotic co-assembly of the betaine-loading ZIF-8 nanocarrier ZSBet and the rhizosphere probiotic *N. capsulatum* based on the polysaccharide-binding protein SPBP. This co-assembly could be targeted and colonized in the wheat rhizosphere regions, simultaneously achieving the continuous release of betaine. It effectively regulates the rhizosphere microbial community and enriches beneficial bacterial communities with the EPS secretion capacity, thereby helping plants to better cope with the saline–alkali stress. However, it should be emphasized that these findings with the potential impacts were just demonstrated under current field experimental conditions. The long-term performance and environmental safety of this co-assembly strategy still require additional and prolonged field trials before practical application. This study proposes an extensible material–microbial co-assembly strategy, providing a promising path for the development of saline–alkali land amelioration technology.

## Figures and Tables

**Table 1:** Effects of ZSBet, Novo and ZSBet + Novo on the physicochemical properties of wheat rhizosphere soil after 110 days of cultivation. Data are presented as mean ± SD (*n* = 5). Different lowercase letters indicate statistically significant differences among treatments at *p* < 0.05 (one-way ANOVA followed by Tukey’s test).

	Control	ZSBet	Novo	ZSBet + Novo
pH	8.53 ± 0.15 a	8.10 ± 0.10 b	8.23 ± 0.12 bc	7.90 ± 0.10 c
Salt content (%)	0.42 ± 0.02 a	0.39 ± 0.02 ab	0.38 ± 0.02 b	0.31 ± 0.03 c
Organic matter (g/kg)	26.77 ± 1.31 c	28.70 ± 0.92 bc	29.33 ± 1.06 ab	31.00 ± 1.23 a
TN (g/kg)	1.38 ± 0.04 c	1.54 ± 0.06 b	1.55 ± 0.07 b	1.79 ± 0.03 a
Available N (mg/kg)	87.67 ± 5.13 b	92.33 ± 3.06 b	91.33 ± 1.53 b	100.33 ± 4.04 a
Available P (mg/kg)	48.67 ± 2.52 c	54.67 ± 2.52 b	55.33 ± 2.52 b	61.00 ± 2.00 a

## Data Availability

The original contributions presented in the study are included in the article/Supplementary Material. The raw data of microbiome analysis are available from the corresponding author on reasonable request.

## Supplementary Materials

The following supporting information can be downloaded at https://www.mdpi.com/article/10.3390/molecules30183669/s1, Figure S1: The sequence and synthesis process of the designed SPBP. Figure S2: The sizes of the prepared ZIF-8 nanoparticles. Figure S3: Powder XRD patterns of Z0, ZS, and ZSBet after 110 days of storage in air. Figure S4: Betaine-releasing kinetics of ZSBet at different pH values (a) and NaCl concentrations (b). Figure S5: Effect of ZSBet on the viability of the probiotic Novo. Figure S6: Quantification of viable bacteria adhered to the roots treated by the ZIF-8 particles. Figure S7: Quantification of the Zn intensity on the roots treated by the ZIF-8 particles. Figure S8: Zn^2+^ contents in the rhizosphere soils of at the initial (0 day) and after 110 days of cultivation (110 day).
