# Supplementary material for: Synthetic Protein-Assisted Co-Assembly of Zeolitic Imidazolate Framework-8 and Novosphingobium capsulatum for Enhanced Saline–Alkali Resistance of Wheat
Source: Molecules. 2025 Sep 9;30(18):3669. doi: 10.3390/molecules30183669 (PMC12472543; doi:10.3390/molecules30183669)
Supplement: Supplementary file 1 [file molecules-30-03669-s001.zip › molecules-3828404-supplementary.pdf]

Supporting Information

Synthetic Protein-Assisted Co-Assembly of Zeolitic Imidazolate Framework-8 and *Novosphingobium capsulatum* for Enhanced Saline–Alkali Resistance of Wheat

Zirun Zhao, Rou Liu, Jiawen Yu, Yunlong Liu, Mingchun Li and Qilin Yu *

National Key Laboratory of Intelligent Tracking and Forecasting for Infectious Diseases, College of Life Sciences, Nankai University, Tianjin 300071, China
* Correspondence: yuqilin@mail.nankai.edu.cn

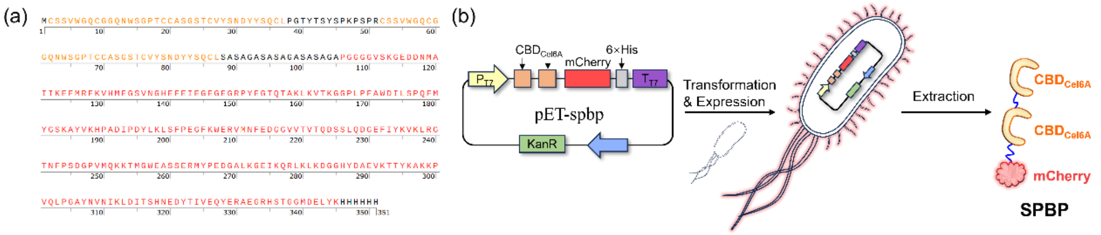

Figure S1. The sequence and synthesis process of the designed SPBP. (a) The sequence of SPBP. The orange and red letters indicate the sequences of CBD_{Cel6A} and mCherry, respectively. (b) A scheme illustrating the construction of the artificial *Escherichia coli EcSPBP* and the following preparation of the protein SPBP.

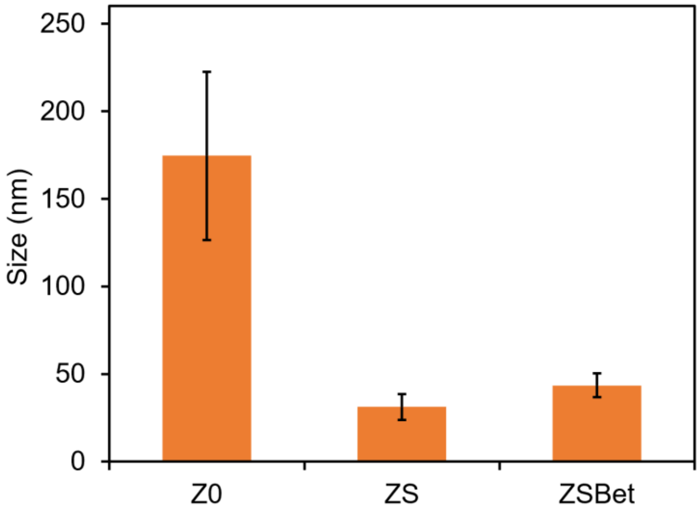

Figure S2. The sizes of the prepared ZIF-8 nanoparticles. The results were shown with the means ± SD.

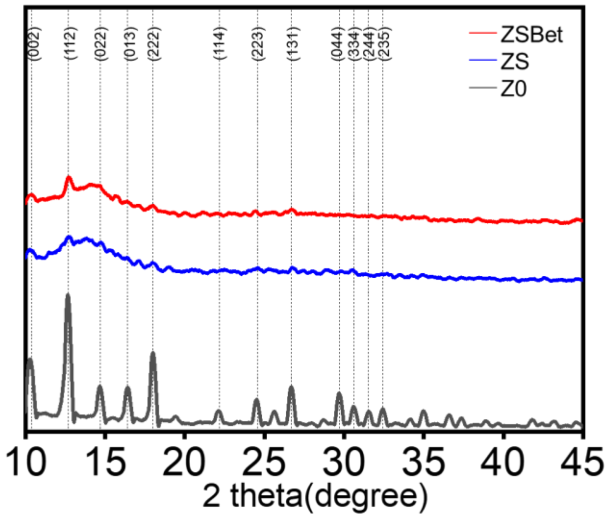

Figure S3. Powder XRD patterns of Z0, ZS, and ZSBet after 110 days of storage in air. The gray curve represents Z0, the blue curve represents ZS, and the red curve represents ZSBet. The curves provide a comparison of the diffraction features of different materials after the long-term storage.

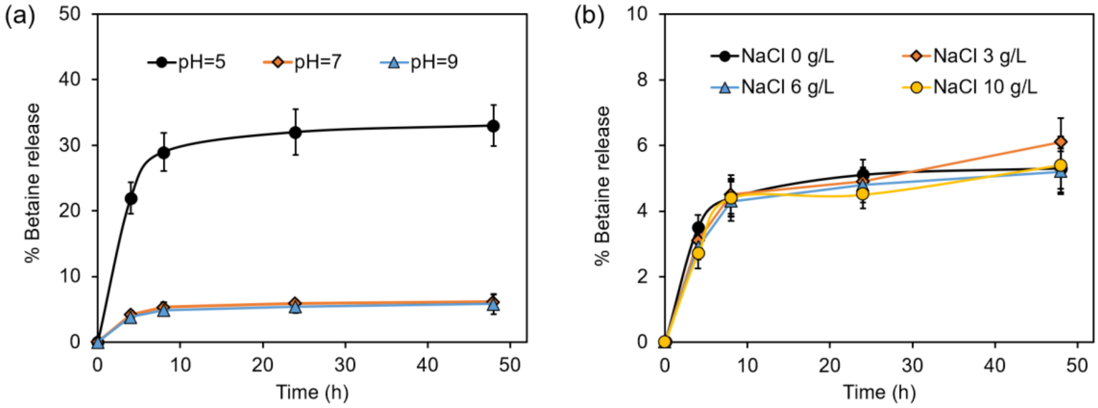

Figure S4. Betaine-releasing kinetics of ZSBet at different pH values (a) and NaCl concentrations (b). The ZSBet particles were suspended to the initial concentration of 1 mg/mL in PBS buffer with the indicated pH values (a), or in the NaCl solutions with different concentrations (b). At different time points of incubation at the room temperature, the suspensions were sampled, centrifuged at 12, 000 rpm for 3 min, followed by quantification of betaine contents in the supernatants with HPLC-MS analysis. The percent of betaine release was calculated by the betaine contents in the supernatants divided by that in the pellets.

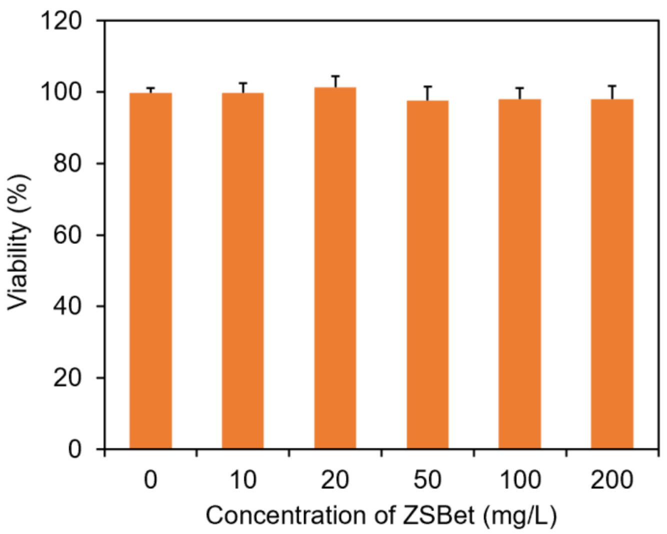

Figure S5. Effect of ZSBet on the viability of the probiotic Novo. The Novo cells were treated by ZSBet at different concentrations for 6 h, followed by CFU assays. The percent of viability was calculated by the CFUs of the treated groups divided by the CFUs of the control group (i.e., the "0" group). Noting that there is no significant difference between the groups ($P < 0.05$, one-way ANOVA followed by Tukey's test).

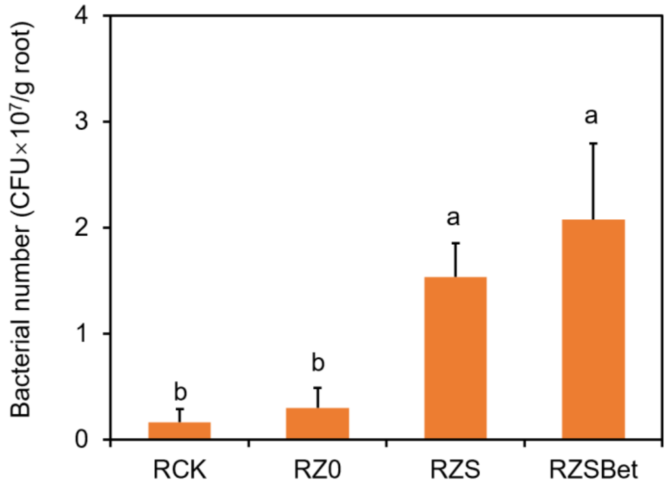

Figure S6. Quantification of viable bacteria adhered to the roots treated by the ZIF-8 particles. Different lowercase letters indicate statistically significant differences among treatments at $P < 0.05$ (one-way ANOVA followed by Tukey's test).

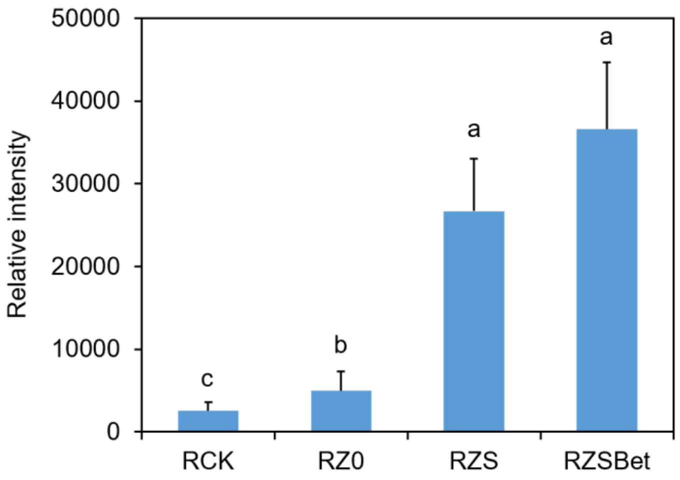

Figure S7. Quantification of the Zn intensity on the roots treated by the ZIF-8 particles. The Zn intensity was analyzed by the Image J software. Different lowercase letters indicate statistically significant differences among treatments at P < 0.05 (one-way ANOVA followed by Tukey's test).

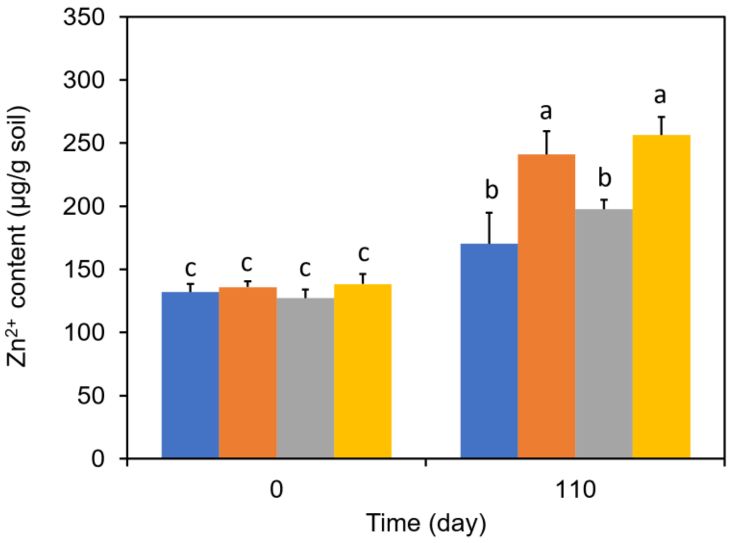

Figure S8. Zn^{2+} contents in the rhizosphere soils of at the initial (0 day) and after 110 days of cultivation (110 day). Different lowercase letters indicate statistically significant differences among treatments at P < 0.05 (one-way ANOVA followed by Tukey's test).
